# Muscle fiber conduction velocity *in situ* revisited: A new approach to an ancient technique

**DOI:** 10.3389/fneur.2023.1118510

**Published:** 2023-02-23

**Authors:** João Aris Kouyoumdjian, Carla Renata Graca

**Affiliations:** Neuromuscular Investigation Laboratory, Department of Neurological Sciences, Psychiatry and Medical Psychology, State Medical School (FAMERP), São José do Rio Preto, São Paulo, Brazil

**Keywords:** muscle fiber conduction velocity, muscle fiber electrical activation, jitter, muscle fiber action potential, velocity recovery function, single-fiber electromyography

## Abstract

The aim of this study was to measure the muscle fiber conduction velocity (MFCV) *in situ* in the tibialis anterior muscle in healthy subjects. A total of 36 subjects matched for age and sex were studied. The MFCV was measured with a concentric needle by intramuscular monopolar needle electrical activation at a distance of 50 mm. The mean consecutive difference (MCD) of <5 μs was obtained after a median of 62 muscle fiber action potentials (MFAPs), confirming a direct muscle fiber activation. The measuring latency was at the median point of ascending depolarizing line of the MFAP. The calculated MFCV from 784 MFAPs was 4.10 ± 0.66 m/s, 3.99 ± 0.57 for female subjects (95%, 2.85 to 5.13), and 4.20 ± 0.73 for male subjects (95%, 2.74 to 5.67). The MFCV was 5.22% faster in male subjects. The calculated fast-to-slow MFCV ratio (F/S ratio) was 1.47 for female subjects (95%, 1.27 to 2.54) and 1.67 for male subjects (95%, 1.31 to 3.74). Aging significantly increased the F/S ratio. As the MFCVs mainly depend on the muscle diameter, their assessment is a quick and helpful tool for estimating it. Its variability by the F/S ratio is also a powerful tool in the follow-up of some neuromuscular disorders.

## Introduction

The propagation of the action potentials of isolated striated human muscle fibers *in situ*, also referred to as the muscle fiber conduction velocity (MFCV), was well described by Buchthal et al. (1, 2) and Stålberg (3).

Buchthal et al. (1, 2) used electrical stimulation by a bipolar electrode in the biceps brachii muscle, eliciting rectangular pulses of 60–100 μs. A concentric needle electrode (CNE) recorded muscle fiber action potentials (MFAPs) distally from the stimulating electrode at three points simultaneously with a distance of 10–35 mm for the first, 15–35 mm for the second, and 15–35 mm for the third. They considered the potentials with a 2–4 ms duration and 20–200 μV amplitude representing isolated muscle fibers. The MFCV found was 4.02 ± 0.13 m/s (1) and 4.10 ± 0.15 m/s (2).

Stålberg (3) did the MFCV studies in the biceps brachii, extensor digitorum, quadriceps femoris, and frontalis muscles using a 1+13 multielectrode in a small port compared with the large one used by Buchthal et al. (1, 2). It was inserted into the muscle at a fixed distance. After intramuscular electrical stimulation away from the motor point, he calculated the time for the action potential passage on each port. Moreover, only the morphological aspects define an MFAP, and he was not always sure about direct or indirect activation. The MFCV found for the biceps brachii muscle was 3.69 ± 0.71 m/s. The influence of the time interval from the preceding action potential on the next propagation velocity, the so-called velocity recovery function (VRF) effect, was thoroughly discussed.

With the progress of digital technology and so many improvements in signal-to-noise machines, some difficulties were overcome and kept in the past. The MFCV *in situ* was replaced by surface electromyography (EMG) recording after a voluntary contraction, which is more accessible and painless. The waves of muscle contraction were recorded externally in the skin by trips in well-defined spaced intervals. However, this method measures the velocity of the motor unit action potential (MUAP) propagation and obeys the Henneman principle (4).

The primary purpose here is to revisit the MFCV *in situ* with some modifications using the same principles described by Troni et al. (5) in healthy subjects to get reference values for the tibialis anterior (TA) muscle. We describe a fast, reliable, and relatively painless technique. The 100% confirmation of isolated muscle fiber action potentials (MFAPs) is now possible based on the mean consecutive difference (MCD) of as many as a hundred MFAPs.

## Methods

The study was in accordance with the Helsinki Declaration of 1975 and approved by the ethics committee of the Faculdade de Medicina de São José do Rio Preto, São Paulo, Brazil, where the tests were performed. All patients signed informed consent.

### Subjects

A total of 36 healthy adult subjects of equally distributed sexes were recruited and invited to participate in the study.

### Electrophysiological material

A Natus machine with UltraPro™ S100 Elite software in Synergy^®^ mode (*Neurodiagnostic System, Middleton, WI, USA*) was used in all subjects. The recordings were performed using a “facial” CNE 25 mm x 30G with a recording area of 0.020 mm^2^ (*Dantec*^®^ *DCN, Natus Manufacturing Limited, Ireland*). An amplitude detection algorithm was used to record and analyze the MCD values. The intramuscular electrical stimulation was delivered by a disposable monopolar needle electrode (cathode), 25 × 0.36 mm, and 28 G (*Ambu*^®^*, Neuroline, Malaysia*). For the reference electrode (anode), an adhesive electrode was used ~1–2 cm away from the cathode.

### Variables

The age, sex, leg temperature, room temperature, and body mass index (BMI) of the 36 subjects were recorded as one measure per subject. The temperature was measured on the anterior leg and kept above 30°C. The room temperature was kept above 22°C.

The number of MFAPs and the MFCV values was estimated to be 20 per subject.

The MFAP has confirmed after 50–100 consecutive discharges a time variability (MCD) of < 5 μs, similar to the number used for the neuromuscular jitter (6). However, < 50 could be acceptable to confirm a direct muscle fiber stimulation.

**Figure 1:**
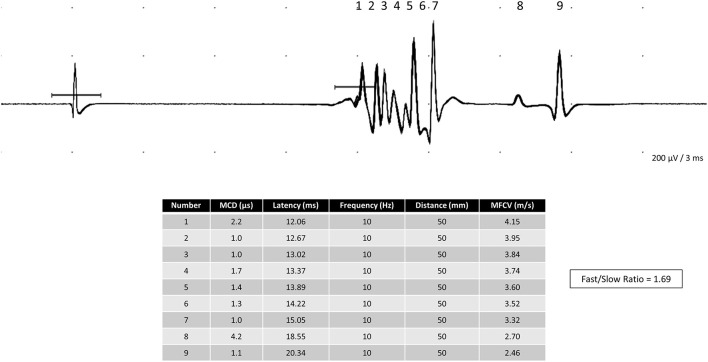
Polyphasic complex of nine single muscle fiber action potentials in the tibialis anterior was obtained after the electrical stimulation of an intramuscular monopolar needle. The fast-to-slow muscle fiber conduction (F/S ratio) calculated in this complex is 1.69.

The mean MFCV value, the fastest divided by the slowest MFCV from each subject (F/S ratio), and the semi-quantitative MUAP amplitude values obtained by needle EMG were one value per subject. An example of the F/S ratio calculation is shown in Figure 1.

Depending on the normality test, some variables have mean or median values.

### Tibialis anterior muscle motor point reference

The endplates in the TA muscle are not sharply demarcated and are distributed along the whole muscle by staining longitudinal cryosections for cholinesterase (7). Despite the staining motor point demarcation, other descriptions, using an electrophysiological analysis, found the main motor point located at the proximal third of the TA muscle belly (8). Botter et al. (9) found another minor motor point located distally and laterally between the middle and the distal third. The main motor point could be localized by Buchthal et al. (1) tracing a line between the tibial bone tuberosity at the knee down to a median line between the two malleoli; the limit between the upper and the middle third is the reference point and (2) tracing a line between the fibular head and the medial malleolus; the limit between the upper and the middle third is the reference point (10).

### Muscle fiber stimulation

All subjects had the right TA muscle studied.

Before starting the test, a semi-quantitative needle EMG was conducted to evaluate the MUAP morphology. We did a 20-s MUAPs recording with mild contraction and measured the mean amplitude (μV) by adding two lines above and below this 20-s recording. If either myopathic or neurogenic abnormalities in the MUAPs were found, the subject was unsuitable for the reference values calculation. The same is true if fibrillation and positive sharp wave potentials were present.

**Figure 2:**
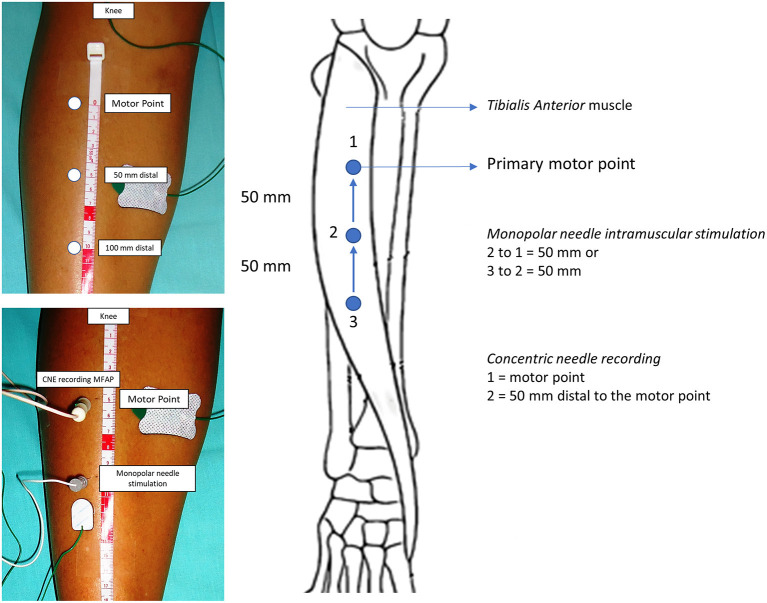
Tibialis anterior muscle anatomical landmarks for needle insertion. The recorded concentric needle electrode (CNE) is inserted in the motor point or 50 mm distal. The monopolar stimulation needle electrode is inserted 50 mm distal to the recording CNE. The muscle fiber conduction velocity is reliably calculated if both stimulation and recording needles are inserted and kept at a right angle.

The first author (JAK) did all tests. The recorded CNE was inserted into the TA muscle at the main motor point. The monopolar stimulation needle is inserted 50 mm distal to the motor point (5, 11) in a straight line traced just lateral to the tibial bone border. Care was taken to maintain both needle electrodes at a right angle so that the distance could be as reliable as possible (Figure 2). If this first attempt did not give rise to MFAPs, the recording and the stimulation needles were moved further 50 mm distally (Figure 2). A 10-Hz pulse stimulus and 0.05–0.10 ms duration were delivered with gently increased intensity in 0.10 mA steps until SFAP recording mostly reached ~0.5–2 mA.

A motor axon stimulation can occur during the test, originating neuromuscular jitter, easily identified by the MCD >5 μs and by random instead of straight dots in the latency *vs*. response graph. The stimulation and recording needle electrodes in the TA muscle should be kept as superficial as possible. For getting the direct stimulated isolated MFAPs, the examinator often does not see, and the subject does not feel the muscle twitch. If visible and felt, there is a high probability of getting a neuromuscular jitter (10). Filters were set at 1,000 Hz and 10 kHz. The sweep was kept at 5 ms/division and the gain to 0.2 mV/division. When both needles reach the muscle fibers, one for stimulation and the other for recording, we usually see a polyphasic complex of many MFAPs with a very short rise time. They appear mostly at ~9–15 ms. The polyphasic complex of many MFAPs usually appears closest, and we should change the sweep to split them apart.

### Muscle fiber conduction velocity measurement

**Figure 3:**
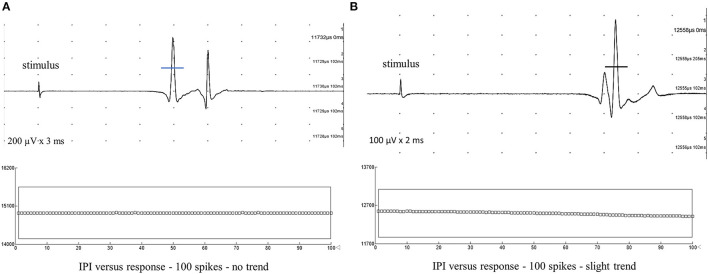
Typical muscle fiber action potentials after intramuscular monopolar needle electrode stimulation were recorded from the tibialis anterior muscle with five superimposed traces. The mean consecutive difference of <5 μs after 100 spikes confidently indicates direct muscle fiber stimulation. The latency mark is made in the middle of the depolarizing negative line to calculate muscle fiber conduction velocity (MFCV). **(A)** Latency = 11.73 ms. MFCV = 4.24 m/s, no trend. **(B)** Latency = 12.42 ms. MFCV = 4.03 m/s. Due to the slight trend in the graph interpotential interval (IPI) or latency vs. response, the MFCV should be calculated to the shortest latency.

**Figure 4:**
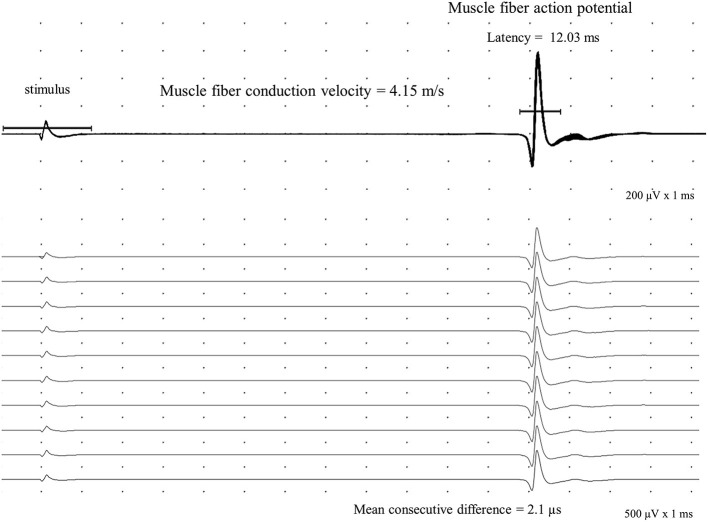
Direct muscle fiber action potential (MFAP) is recorded in the tibialis anterior with a concentric needle electrode after an intramuscular monopolar needle electrical stimulation at 10 Hz with a 50 mm distance. The mean consecutive difference with a 2.1 μs value in a sequence of a hundred potentials (shown 10) defines a direct muscle activation. The calculated muscle fiber conduction velocity is 4.15 m/s.

**Figure 5:**
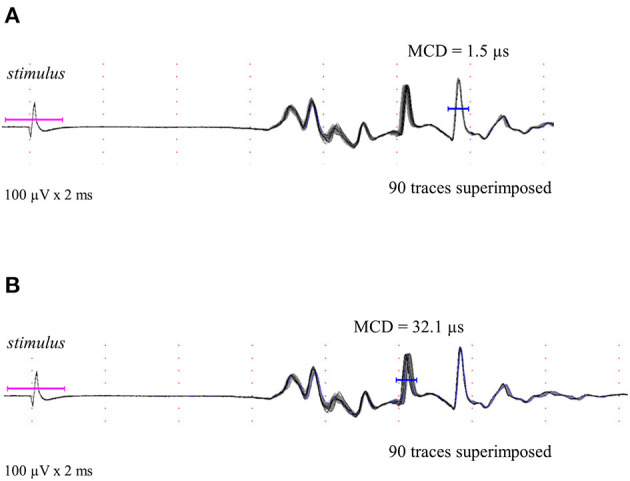
Polyphasic complex of some muscle fiber action potentials after stimulation is shown as described in methods. Different muscle fiber action potentials could be obtained by direct and indirect stimulation. **(A)** Typical direct stimulated muscle fiber action potential is shown and confirmed by the mean consecutive difference superimposed (90 traces and 1.5 μs). **(B)** Typical indirect stimulated muscle fiber action potential (neuromuscular jitter) is shown and confirmed by the mean consecutive difference superimposed (90 traces and 32.1 μs).

We chose the TA muscle as it is long and has parallel fibers allowing direct muscle stimulation many centimeters away from the motor point. Moreover, it has reliable external marks to measure the distance (and, consequently, the MFCV) and produces minor pain. We chose the shortest latency value as sometimes we have slight trends (Figure 3). The MFAP latency measurement for MFCV calculation was done in the middle of the ascendent depolarizing line of the MFAPs (Figure 4) so that the neuromuscular jitter could be immediately identified and distinguished from the direct muscle fiber stimulation (Figure 5). As the distance is fixed, the MFCV was obtained as 50 mm/latency in milliseconds.

Mostly, we did three needle insertions, always moving both electrodes and keeping both in the same straight line. It is unnecessary to keep increasing the stimulus intensity, as the MFAPs had an easily seen all-or-none response. The F/S ratio was calculated for each subject (Figure 1).

### Statistics

Descriptive statistics: mean, median, standard deviation (SD), and percentiles were calculated for the continuous variables. Normality tests: Anderson–Darling, D'Agostino and Pearson, Shapiro–Wilk, and Kolmogorov–Smirnov (confidence interval = 95%). The upper or lower limit of normality for the variables was defined as mean and SD (normal distribution) and as the 5 and 95% values (non-normal distribution). The *p*-value was set to *p* < 0.05. Variables comparison: (1) Parametric unpaired Student's *t*-test for normally distributed variables. (2). Nonparametric Mann–Whitney U-test and the Kolmogorov–Smirnov for non-normally distributed variables. The comparison tests were set to a 95% confidence interval. Correlation coefficients (r): (1) Pearson-r for measuring the strength of the linear relationship between two parametric variables (range, −1 maximum negative, 0 no correlation, and +1 maximum positive). (2) Coefficient of determination (R-squared) for the variance of two variables, ranging from 0 (no correlation) to 100% (perfect correlation). (3) Nonparametric Spearman-r (rho) ranging from −1 to +1. Software: All calculations were performed using Minitab^®^ Statistical Software (*State College, Pennsylvania, USA) and Excel*^®^ *(Microsoft Office, Redmond, Washington, USA*).

## Results

### Patients

There were 18 male subjects and 18 female subjects. The mean age of the 36 subjects was 41.11 ± 12.34 years (21–60), 43.44 ± 12.51 years (23–60) for male subjects, and 38.78 ± 12.05 years (21–57) for female subjects. None of the subjects were using anticoagulants. None of them had diabetes mellitus, neuromuscular comorbidities, or neurogenic/myopathic electromyographic findings in the TA muscle. None of them had medical conditions that could cause disuse.

### Variables

The mean or the median of nine variable values—age, number of MFAPs, MCD, MFCV, F/S ratio, BMI, MUAP amplitude, leg temperature, and room temperature—obtained from the 36 subjects is shown in Table 1. The median values and the lower and upper limits (α = 0.05) for the primary dependent variable F/S ratio and sex are shown in Table 2. The MFAP was confirmed by an MCD of < 5 μs obtained from a median of 62 consecutive discharges (percentile 25% = 33 discharges and percentile 75% = 100 discharges).

**Table 1:** Mean or median from nine variables from the 36 subjects, 18 female subjects and 18 male subjects.

	**Age**	**nMF**	**MCD**	**MFCV**	**F/S ratio**	**BMI**	**MUAP**	**Leg T**	**Room T**
Normality test	yes	no	yes	Yes	No	yes	no	yes	yes
Number of values	36	36	36	36	36	34	32	31	31
Minimum	21	10	2.2	3.3	1.27	18.5	1,256	29.4	22.1
5%	21.8	10.8	2.4	3.4	1.29	19.9	1,329	29.8	22.5
Median	42	22.5	3.2	4.0	1.62	27.5	1,698	32	24
Maximum	60	27	4.0	5.0	3.74	40.6	3,438	33.6	26.9
95%	58.3	27	3.9	4.9	3.15	38.9	3,302	33.1	26.7
Mean	41.11	21.78	3.24	4.10	1.73	27.94	1,923	31.87	24.49
Std. deviation	12.34	3.98	0.44	0.37	0.49	5.68	616.1	0.88	1.29

nMF, number of muscle fibers; MCD, mean consecutive difference (μs); MFCV, muscle fiber conduction velocity (m/s); BMI, body mass index; MUAP, the amplitude of the motor unit action potential (μV); Leg T, leg temperature (°C); Room T, room temperature (°C); F/S ratio, fast-to-slow muscle fiber conduction velocity.

**Table 2:** Median and reference limits for the F/S ratio variable and sex.

	**F/S ratio**	**F/S ratio**	**F/S ratio**
Sex	Both	Female	Male
N	36	18	18
Median	1.62	1.47	1.67
Lower limit	1.27	1.27	1.31
Upper limit	3.74	2.54	3.74

MFCV, muscle fiber conduction velocity (m/s); F/S ratio, fast-to-slow muscle fiber conduction velocity; F, female subjects; M, male subjects.

### Muscle fiber conduction velocity

The MFCV was calculated from the individual values and it is shown in Table 3. The total number of MFAPs recorded was 784, with a median of 22.5 per subject. The MFCV was 5.22% faster in male subjects than in female subjects.

**Table 3:** Mean and reference limits of MFCV from 784 individual values.

	**MFCV all**	**MFCV female**	**MFCV male**
Muscle fiber action potentials	784	369	415
Mean	4.108	3.997	4.206
Standard Deviation	0.6691	0.5707	0.7324
Lower limit	2.7698	2.8556	2.7412
Upper limit	5.4462	5.1384	5.6708

MFCV, muscle fiber conduction velocity (m/s).

**Figure 6:**
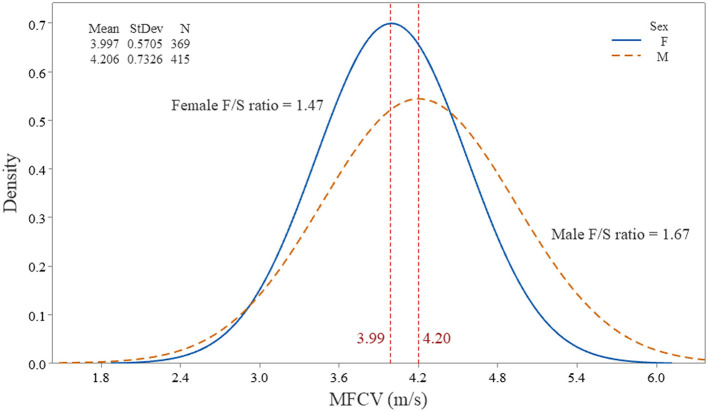
Histogram of the individual muscle fiber action potentials (784) from 36 subjects and its distribution, according to the muscle fiber conduction velocity (MFCV). Note the female and male subjects on separate curves. The fast-to-slow MFCV ratio (F/S ratio) was higher in male subjects, indicating a more significant muscle fiber diameter variation.

**Figure 7:**
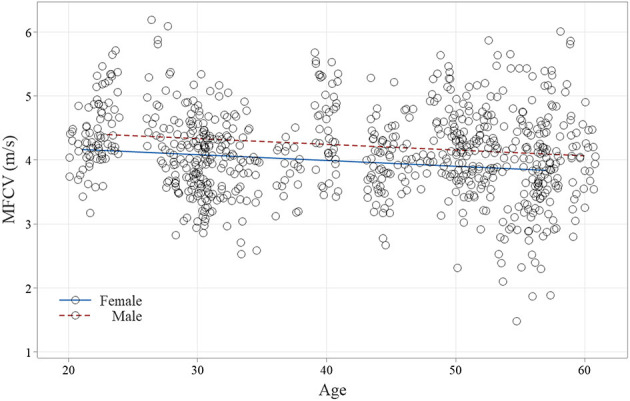
Scatterplot of the muscle fiber conduction velocity (MFCV) from 784 muscle fiber action potentials (female subjects, 269 and male subjects, 415) is displayed throughout the age. Note that both sex lines had a small R-Sq and ran in parallel, maintaining the faster velocity in male subjects throughout the aging.

The histogram of the MFCV and age in male and female subjects is shown in Figure 6. It is worth noting that the bell-shaped distributions for both sexes, as well as a more spread curve for male subjects, indicate a slightly higher F/S ratio, 1.67 vs. 1.47. The scatterplot of individual MFCV values *vs*. the categorized sex and the continuous age variables are shown in Figure 7. The MFCV of male and female subjects decreases slightly in parallel over the years, faster in male subjects by about 5%.

### Correlation

The Pearson correlation coefficient (r) revealed that the leg temperature (0.10% and *p*-value = 0.8559) and BMI (0.27% and *p*-value = 0.7804) did not influence the MFCV (not significant). Age had a negligible (not significant) correlation with the MFCV decreases (4.26%, and *p*-value = 0.2270).

The Spearman correlation coefficient (r) revealed: 1. The mean number of MFAPs obtained per subject (22.5) did not vary together with the MFCV (Spearman-r = 0.007054 and *p*-value = 0.9674); 2. The MUAP amplitude has a slight, but no significant, inverse correlation to the MFCV (Spearman-r = −0.2900 and *p*-value = 0.1074); 3. The F/S ratio strongly correlates to age (Spearman-r = 0.5613 and *p*-value = 0.0004).

## Discussion

### Main findings

Our results revealed an MFCV of 4.10 ± 0.36 m/s, 4.22 m/s for male subjects and 3.99 m/s for female subjects. The lower and upper limits for the neurophysiological practice were 2.76–5.44 m/s, 2.74–5.67 m/s for men, and 2.85–5.13 m/s for women. Compared with the one from Troni et al. (5), our new method had the following changes: (1). A thin “facial” (30G) CNE is used for recording. (2) A 10-Hz electrical stimulation is delivered for the MFAP acquisition. (3) The MCD < 5 μs was obtained after a median of 62 consecutive MFAPs. (4) The time variation is done by the amplitude, using a bar mark in the middle of the ascending depolarizing line of the MFAP, being highly reliable. (5) The calculated MFCV is taken from the MFAP with the shortest latency. (6) The MFAPs dots on the histogram are easily selected, eliminating the spurious array. (7) The response *vs*. latency graph makes distinguishing direct or indirect muscle stimulation easy. (8) The after-exam edition considerably shortens the test. (9) The polyphasic complex with many MFAPs is automatically recorded as a video file. (10) For getting the reference values, the TA muscle MUAPs must have normal morphology to exclude neurogenic and myopathic conditions that may have extensive muscle fiber diameter variability.

### Muscle fiber conduction velocity

We retrieve the MFCV *in situ* as described earlier. The stimulation reaches both fiber types, I and II, randomly. Most of the articles (1–3, 5, 12–18) reported just 1, 2, or 5 MFAPs not enough to get a reliable MCD value that defines a direct muscle stimulation. Moreover, the measurement for MFCV calculation was done mainly at the first positive or negative deflection or the peak latency (5, 15, 19, 20). As the rise time for an MFAP is very short (75–200 μs) (21), the MFCV varied slightly, shorter when measured for the first deflection latency or longer for the peak latency. Table 4 summarizes the findings in some reports.

**Table 4:** Summary of the main reports on muscle fiber conduction velocity *in situ*.

**Author**	**Year**	**Muscle**	**Recording**	**MFCV (m/s)**
Buchthal et al.	1955	*Biceps Brachii*	CNE	4.02 ± 0.45
Stålberg	1966	*Biceps Brachii*	Multielectrode	3.69 ± 0.71
Stålberg	1966	*Extensor Digitorum*	Multielectrode	3.15 ± 0.75
Stålberg	1966	*Quadriceps*	Multielectrode	3.39 ± 0.68
Stålberg	1966	*Frontalis*	Multielectrode	2.01 ± 0.39
Troni et al.	1983	*Biceps Brachii*	SFE	3.81 ± 0.34 (M)
Troni et al.	1983	*Biceps Brachii*	SFE	3.42 ± 0.33 (F)
Chino et al.	1984	*Biceps Brachii*	Monopolar Electrode	5.10 ± 0.80
Zwarts	1989	*Biceps Brachii*	CNE	3.20 ± 0.30 (M)
Zwarts	1989	*Biceps Brachii*	CNE	3.10 ± 0.40 (F)
van der Hoeven et al.	1994	*Biceps Brachii*	CNE	3.17 m/s
Naumann and Reiners	1996	*Rectus Femoris*	CNE	4.00 m/s
Al-Ani et al.	2001	*Biceps Brachii*	CNE	3.59 ± 0.32
Al-Ani et al.	2001	*Tibialis Anterior*	CNE	3.66 ± 0.35
Vogt and Fritz	2006	*Biceps Brachii*	CNE	3.36 ± 0.20
Methenitis et al.	2018	*Vastus Lateralis*	CNE	5.25 ± 0.64
Present study	2022	*Tibialis Anterior*	CNE	4.20 ± 0.73 m/s (M)
Present study	2022	*Tibialis Anterior*	CNE	3.99 ± 0.57 m/s (F)

CNE, concentric needle electrode; SFE, single-fiber electrode; MFCV, muscle fiber conduction velocity; M, male subjects; F, female subjects.

The MFCV increases with the muscle diameter from childhood to adulthood (21), but it is a linear and not a proportional correlation. It should be pointed out that despite the increase in the muscle fiber diameter by a factor of 5 between 1 and 30 years, the MFCV was independent of the age of the subjects (1, 2). Our results in adults, like others (1, 2, 20–22), showed a slight correlation between age and MFCV decreases.

Our findings showed an MFCV of 5.2% less in female subjects, similar to that described by Troni et al. (5) and Graham et al. (23). Adult female subjects have a smaller muscle fiber diameter than male subjects (24). Therefore, a lower MFCV is expected in female subjects. Naumann and Reiners (22) did not disclose any difference. As the MFCV had an almost perfect normal distribution, we can estimate the usual minimum and maximum diameter with 95% confidence. The estimated muscle fiber diameter by knowing the MFCV in healthy humans was projected by the formula MFCV (m/s) = 0.05 x muscle fiber diameter (μm) + 0.95 (25).

**Figure 8:**
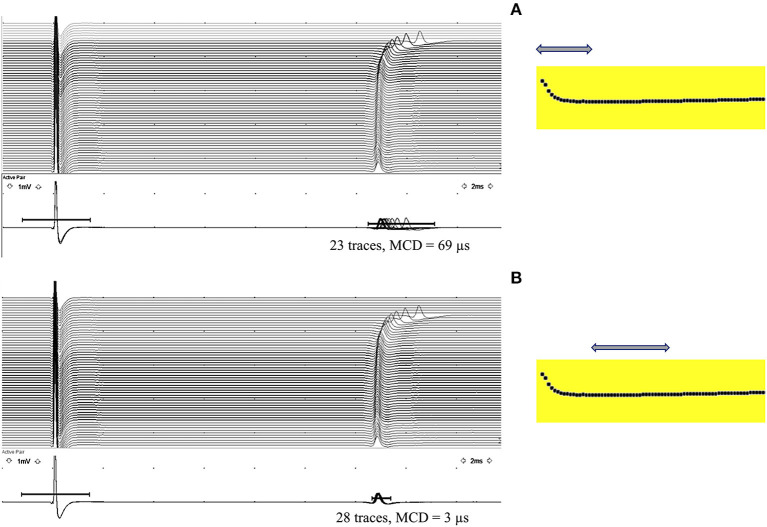
Velocity recovery function (VRF) is typically seen in the first second of a 10-Hz electrical activation. **(A)** Observe a progressive latency reduction in the first 10 muscle fiber action potentials (MFAPs), with a mean consecutive difference (MCD) of 69 μs measured from 23 MFAPs (arrow). **(B)** After the initial latency reduction, stabilization occurs with an MCD of 3 μs measured from 28 MFAPs (arrow), compatible with a direct muscle fiber activation.

The stimulation rate enhancement increases the MFCV. From 5, 10, 15, and 20 Hz, the MFCV increased by 0.03, 0.15, 0.30, and 0.40–0.60 m/s, respectively (3). From 5 to 20 Hz, the MFCV increased by 5–13.3% (15). Most of the studies used 1-Hz stimulation to obtain just 1–5 MFAPs. Here, we use a 10-Hz electrical activation, simulating better a physiological condition and also being the test faster. The MFCV values obtained here should be 8–10% higher than most studies. The MFCV can be affected by the VRF when the firing rate changes during the electrical activation (26), and care was taken to avoid it by seeing the graph in the first second of activation (Figure 8).

### F/S ratio

One of the significant advantages of measuring MFCV *in situ* is a reliable calculation for the first and last MFAPs representing the F/S ratio (15, 16, 18, 21, 27). The F/S ratio found here was 1.62, comparable to 1.65 from Allen et al. (27). For the biceps brachii muscle, Zwarts (15) found 1.46. For the vastus lateralis muscle, Methenitis et al. (18) found 2.55. The F/S ratio was more in male subjects (1.67 vs. 1.47). Most adult male and female subjects had a muscle fiber diameter ranging between 40–80 and 30–70 μm, respectively (24).

We found that aging significantly increases the F/S ratio. The aging decreases the number of motoneurons, leading to the motor units' reorganization and muscle fiber diameter variability (28). The aging also is associated with type-II muscle fiber atrophy (29), disuse from decreased physical activity, and altered hormonal status (28).

### Clinical utility

The F/S ratio provides critical information about muscle fibers' diameter distribution, as the MFCV varies linearly to them (1–3, 5, 18, 30). The F/S ratio could be helpful in the follow-up of myopathic or neurogenic disorders reflecting the increase or decrease of muscle diameter variability. A marked MFCV slowing (2.24 m/s) was observed in a denervated biceps brachii muscle (5). Atrophic fibers have a significant MFCV reduction relative to the standard muscle fibers (31). The relationship between the reduced muscle fiber diameter and the reduced MFCV was significantly higher in the neurogenic group compared with the myopathic group (20, 32). In patients with inflammatory myopathies, there is a significant MFCV variation, increasing the F/S ratio (15); in acute inflammatory myopathies cases, there is a significant reduction in the MFCV (22). The MFCV had a 12–26% decrease in the vastus lateralis, vastus medialis, and TA muscles in steroid myopathy from Cushing's syndrome (33). The needle EMG has a limited value in steroid myopathy as the first motor unit recruited is composed of type-I fibers, which are less affected in this myopathy (33). Disuse does not alter MUAP characteristics or muscle membrane excitability (34). The MFCV directly correlates to sarcolemmal excitability (34, 35). In critical illness myopathy, muscle fibers can be electrically unexcitable. Allen et al. (27) found a significant MFCV reduction in critical illness myopathy (if excitable). In the TA muscle, patients had a mean MFCV of 2.32 ± 1.12 m/s, in contrast to 4.02 ± 0.60 m/s in controls. As the MFCV depends on excitatory membrane conductance, its reduction is reported for patients with hypokalemic periodic paralysis in attack-free intervals, despite being more pronounced during paralytic attacks (36). In peripheral neuropathy, the muscle fibers retain normal electrical excitability even if entirely denervated (34).

## Conclusion

The muscle fiber conduction velocity (MFCV) *in situ* was measured using the same parameters for the neuromuscular jitter measured with a CNE but changing landmarks for activation and recording in the TA muscle. An intramuscular monopolar needle was inserted 50 mm away from the motor point for the electrical activation, where a CNE was inserted for recording. A median of 62 MFAPs with an MCD of < 5 μs was obtained. The time variation and the latency point for measuring the MFCV were calculated from the median point of the MFAP ascending depolarizing line. The calculated mean MFCV for the TA muscle in 36 subjects and 784 MFAPs were 4.10 ± 0.66 m/s, 4.20 ± 0.73 for male subjects (95% limit and 2.74–5.67), and 3.99 ± 0.57 for female subjects (95% limit and 2.85–5.13). The MFCV was 5.22% faster in male subjects. The calculated F/S ratio was 1.67 for male subjects (95% limit and 1.31–3.74) and 1.47 for female subjects (95% limit and 1.27–2.54). Aging significantly increases the F/S ratio.

## Data availability statement

The raw data supporting the conclusions of this article will be made available by the authors without undue reservation.

## Ethics statement

The studies involving human participants were reviewed and approved by the Ethics Committee of the Faculdade de Medicina de São José do Rio Preto, São Paulo, Brazil and carried out in accordance with the Declaration of Helsinki 1975. All patients signed their informed consent. The patients/participants provided their written informed consent to participate in this study.

## Author contributions

JK designed the study, prepared the protocol, and carried on the muscle fiber conduction velocity tests. CG selected the subjects, tabulated data, and helped in the revision of the manuscript. Both authors participated in the interpretation of data, read, and approved the final manuscript.
